# Efficient In Vivo Introduction of Point Mutations Using ssODN and a Co-CRISPR Approach

**DOI:** 10.1186/s12575-020-00123-7

**Published:** 2020-07-14

**Authors:** Tgst Levi, Anna Sloutskin, Rachel Kalifa, Tamar Juven-Gershon, Offer Gerlitz

**Affiliations:** 1 Department of Developmental Biology and Cancer Research, Faculty of Medicine, Institute for Medical Research Israel-Canada, Hebrew University, Jerusalem, Israel; 2 The Mina and Everard Goodman Faculty of Life Sciences, Bar-Ilan University, Ramat Gan, Israel

**Keywords:** CRISPR/Cas9, co-CRISPR, ssODN, HDR, Point mutations, *Drosophila*, *Nup107*, *tin*

## Abstract

**Background:**

The generation of point mutations is a major tool for evaluating the roles of specific nucleotides or amino acids within the regulatory or functional landscape. However, examination of these mutations in vivo requires the generation of animals carrying only the relevant point mutations at the endogenous genomic loci, which is technically challenging. The CRISPR-Cas9 based genome editing greatly facilitates the generation of such genetically modified animals; however, most of the described methods use double-strand DNA (dsDNA) as the donor template. The dsDNA plasmids frequently undergo undesired integration events into the targeted genomic locus. The use of a single-strand oligodeoxynucleotide (ssODN) as the donor template prevents this complication and is therefore the preferred choice for introducing point mutations, as well as short sequences such as protein tags.

**Results:**

We successfully applied the CRISPR-based *white* co-conversion strategy with a ssODN template, instead of the originally described dsDNA plasmid, to create genetically modified *Drosophila melanogaster* strains. We used the technique to easily introduce point mutations in two distinct chromosomes. Using the generated flies, we were able to demonstrate the in vivo importance of the respective mutations. For the *Nucleoporin107* (*Nup107*) gene, the 1090G > A mutation was confirmed to affect ovarian development, while for the *tinman* (*tin*) gene, the regulatory role of the downstream core promoter element (DPE) was demonstrated within the developing *Drosophila melanogaster* embryo.

**Conclusions:**

The described approach has facilitated the successful generation of point mutations in two different chromosomes, by two different labs. Distinct phenotypes associated with the newly-generated genotype were identified, thus exemplifying the importance of investigating the in vivo role of specific nucleotides. In addition, detailed guidelines, recommendations and crossing schemes are provided in order to support the generation of additional genetically modified animals by the scientific community.

## Background

Point mutations include substitutions, insertions, and deletions of one or more bases, and are a major tool for evaluating the roles of specific nucleotides or amino acids within the regulatory or functional landscape. Mutations of multiple nucleotides are typically used to demonstrate their contribution to promoter or enhancer activity, while single base pair alterations were typically used to demonstrate the function and regulation of enzymes, transcription factors and signal transducers. However, these studies were mainly conducted in vitro, using exogenously provided DNA. Examination of these mutations in vivo, in the context of the whole animal, requires the generation of a genetically modified animal carrying only the relevant point mutations at the endogenous genomic locus. This approach is uncommon, due to its technical challenges, and involving both a long duration (can be years for mice), and a relatively high cost. In addition, a major concern with in vivo studies that introduce point mutations is the presence of genomic scars: restriction enzyme recognition or recombinase recognition sites used to generate the desired modification, which contaminate the genomic sequence and complicate the interpretation of the results. Thus, the in vivo context or relevance of point mutations that were previously analyzed in vitro, often remains unexplored.

The CRISPR/Cas9 (clustered regularly interspaced short palindromic repeat/CRISPR-associated 9) system is a rapidly evolving genome-engineering tool based on the bacterial immune system that was first discovered in 1987. The CRISPR/Cas9 system consists of a guide RNA (gRNA) that recruits the Cas9 nuclease to the target locus in the genome by sequence homology, which then creates double strand breaks (DSBs). The repair of these DSBs can either cause small insertions or deletions (indels) when non-homologous end-joining (NHEJ) repair is used, or can be utilized to introduce specific mutations through a homology-directed repair (HDR) mechanism, by providing a matching donor template flanked by homology arms [1, 2].

CRISPR-based genome editing was adapted for use in both cultured cells and model organisms, and has greatly contributed to the generation of in vivo endogenous mutations in multiple model organisms [3–5]. A key model organism is the fruit fly, *Drosophila melanogaster*, which is widely used for both developmental and disease-related research. Among the fruit fly's major advantages is the ability to analyze genes and phenotypes in vivo, within the context of the entire organism.

CRISPR-directed editing was quickly adapted for the generation of *Drosophila* genetically modified flies harboring various mutations, mostly via knock-out by means of the NHEJ pathway [6, 7]. The use of HDR-directed repair to generate specific mutations is significantly less efficient and thus technically challenging [3]. One elegant approach facilitating the identification of animals with the desired genomic change is the co-conversion strategy, where both the gene of interest and a visible phenotype (eye color) are targeted simultaneously [8]. At present, most of the described methods use dsDNA plasmids as the donor template for the homologous repair, and several reports describe the undesired integration of the plasmid donor backbone (outside of the homology arms) into the targeted genomic locus [8, 9]. Moreover, the frequency of these undesired events greatly differs between the different loci, and is even reported to “vary from 0 to 100%” [10]. The donor plasmid design may also contribute to this variability. Utilizing single-strand oligodeoxynucleotide (ssODN) as the donor template circumvents this complication, due to the nature of the repair mechanism, and therefore ssODN templates should be favored whenever possible. In addition, ssODN templates were shown in the single-cell green alga *Chlamydomonas reinhardtii* to have a dramatically reduced tendency to randomly integrate into the genome, as compared to dsDNA donor templates such as plasmids [11].

Here we describe in detail the application of the CRISPR/Cas9 system using ssODN donor sequences in *Drosophila melanogaster*. We were able to introduce defined point mutations into the *Drosophila* genome, resulting in a single amino acid substitution in the *Nucleoporin 107 (Nup107)* protein or in an alteration of a regulatory motif within the promoter of the *tinman* (*tin*) gene, within as short as one to two months. Notably, the described technique was successfully applied in two different labs, demonstrating the potency of the system.

XX ovarian dysgenesis (XX-OD) is a rare, genetically heterogeneous disorder, in which the patient presents with underdeveloped, dysfunctional bilateral ovaries. The Gerlitz lab recently identified a single missense mutation in the human *Nup107* gene (c.1339G>A, p.D447N) as the causative mutation behind XX-OD in six female relatives [12]. Generating this mutation in *Drosophila* (c.1090G>A, p.D364N) at the endogenous locus will provide new insights into the role of the Nup107 protein, an essential component of the nuclear pore complex (NPC), in oogenesis and ovarian development.

The regulation of transcription is a tightly controlled process regulated by, among other factors, core promoter elements, or short DNA motifs located in close proximity to the transcriptional start site that serve as binding sites for basal transcriptional machinery components [13–15]. One such motif is the downstream core promoter element (DPE), which was shown by the Juven-Gershon lab to regulate *tin* RNA levels in S2R+ cells [16]. Changing the endogenous DPE sequence (7bp within a 5’UTR regulatory region) to a non-functional one will allow the examination of the regulatory effect of this core promoter element on the developing *Drosophila* embryo. Prior attempts to introduce this 7bp mutation in the endogenous locus, using either a ssODN without a selectable marker or a transposon-based approach employing a donor plasmid that contains a visible selectable marker, were unsuccessful.

Thus, an approach that combines the use of a ssODN and a visible selectable marker was desired.

Here, a *white* co-conversion strategy towards CRISPR-directed genome editing was employed [8]. We utilized the flies and the coffee donor plasmid as in the original paper [8]; however, we replaced the suggested dsDNA plasmid with a ssODN as the source of the donor for each gene-of-interest. This novel approach has facilitated the successful generation of point mutations in two different chromosomes, by two different labs. We discuss target site selection and gRNA generation, donor design and construction, and the generation, identification and molecular confirmation of the engineered lines. Using this technique, we can now easily evaluate the in vivo relevance of particular nucleotides or amino acids with minimal cost and within a relatively short time period.

## Results

### General Plan and Major Considerations for Construction of the Transgenic Flies

We applied the *white* co-conversion strategy [8] to introduce point mutations in both the *Nup107* and *tin* genes. To generate these transgenic lines, we used ssODN as the donor template, rather than a double stranded plasmid for the locus of interest, in order to avoid donor integration events.

*White* co-conversion involves the injection of three DNA components into the Cas9-expressing strain (Additional file 1: Figure S1): 1) pCFD4d plasmid harboring two gRNAs targeting the *white* gene and the locus of interest, respectively. 2) pUC57- white [coffee] plasmid serving as a donor template for the *white* locus. 3) A donor template for the locus of interest - ssODN in our case.

**Fig. 1:**
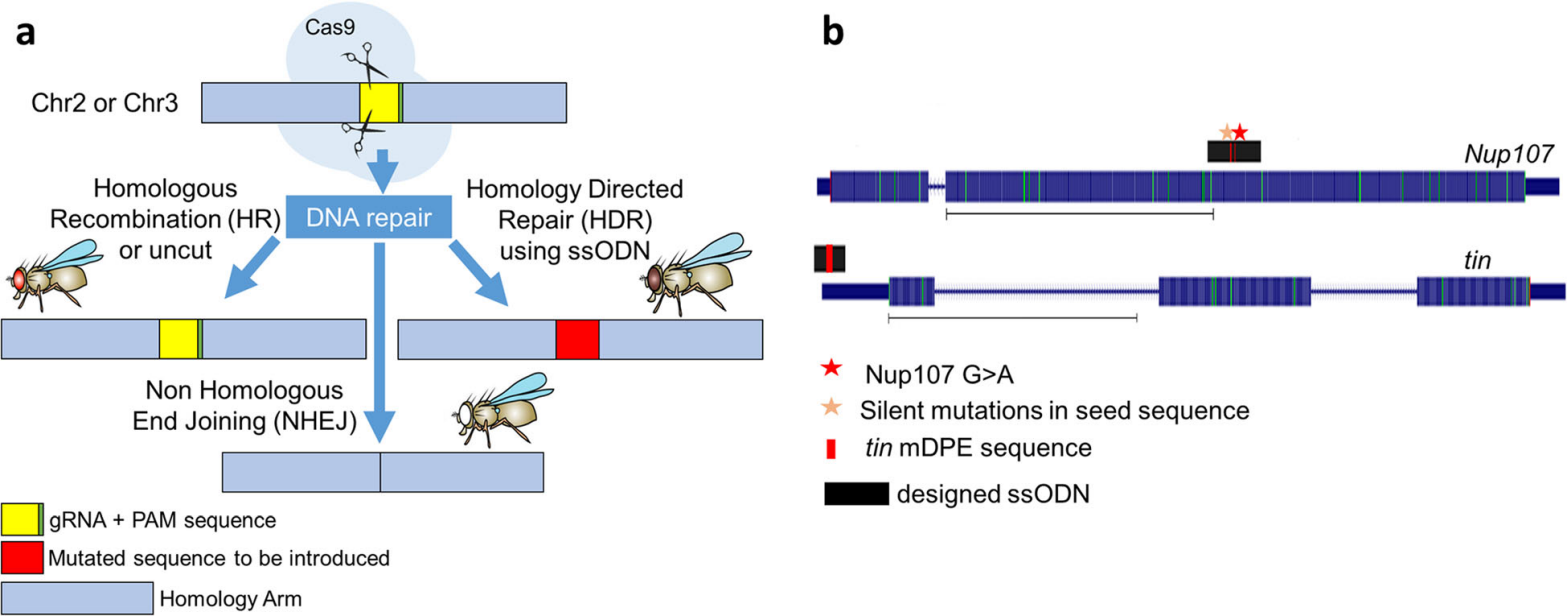
Schematic representation of the co-conversion strategy and the designed mutations. **a** Graphic representation of the original genomic region and possible molecular and phenotypic outcomes, based on *white* co-conversion strategy [8]. Schematic fly images were taken from the *Drosophila* training resources [17]. **b** The designed mutations in *Nup107* and *tin* loci. Both genes are presented according to the UCSC representation, denoting exons, introns and UTR region. Mutations are as indicated in the legend. The scale bar under each gene represents 1Kb

The distinct molecular repair events can be readily distinguished by eye color - red denotes no change (either no initial cutting by Cas9 or the result of a perfect repair), white eyes indicate non-homologous end joining (NHEJ), and coffee (brown) eyes indicate homology-directed repair (HDR) (Fig. 1a). An event of HDR of the *white* gene (visible by coffee-colored eyes) is most likely to occur together with an HDR event of the target gene, thus coffee-eyed flies have high chances of harboring the desired mutation. The ssODN were designed to introduce either distinct missense and silent mutations in the coding region (*Nup107*) or a 7bp sequence altering a transcription-regulatory motif in the 5’ UTR (*tin*), as depicted in Fig. 1b.

For the *white* gene, we used the w gRNA sequences as provided in Table 1 [8]. gRNA design for the gene of interest (*Nup107* or *tin*), was performed using the FlyCRISPR algorithm ([3], http://targetfinder.flycrispr.neuro.brown.edu/). Default parameters were used, and zero off-targets were preferred. In general, the cut site, 3 nucleotides upstream of the protospacer adjacent motif (PAM) sequence, should be as close as possible to the mutation site. It is very important to verify that the gRNA target sequence is the same in the relevant strain using Sanger sequencing of the amplicon derived from the injected strain (~500bp centered at the intended gRNA) - some injection companies offer this as a separate service. Both gRNAs (per gene) were cloned into pCFD4d [7] using either Gibson assembly reaction [18] (*Nup107*) or transfer PCR (TPCR) method [19, 20] (*tin*), as detailed in the methods section. The relevant sequences used for gRNA construction are listed in Table 1.

**Table 1:** Primers and sequences used to construct the pCFD4d gRNA plasmid to create the genetically modified flies

**Name**	**Sequence (5′ to 3′)**
pCFD4d-*tin*_sgRNA_F	TATATAGGAAAGATATCCGGGTGAACTTCGgagctgacaaattgcagacaGTTTTAGAGCTAGAAATAGCAAG
pCFD4d-*w*_sgRNA_R (*tin*)	ATTTTAACTTGCTATTTCTAGCTCTAAAACccaaagagcaggaatggtatcCGACGTTAAATTGAAAATAGGTC
pCFD4d-*w*_sgRNA_F (*Nup107*)	TATATAGGAAAGATATCCGGGTGAACTTCGataccattcctgctctttggGTTTTAGAGCTAGAAATAGCAAG
pCFD4d-*Nup107*_sgRNA_R	ATTTTAACTTGCTATTTCTAGCTCTAAAACtcagatgggcccagagcaaacCGACGTTAAATTGAAAATAGGTC
pCFD4d_sequencing_primer	GACACAGCGCGTACGTCCTTCG
*coffee*_seq_primer	TATCAACGGAGCCATCTTCCTCTTC

The ssODN was planned to introduce the desired changes creating mutation in PAM (*tin*) or with additional silent mutation in the seed sequence (*Nup107*), in order to ablate the Cas9 recognition site to prevent Cas9 from re-cutting the target sequence.

For *Nup107*, the missense mutation to be introduced was c.1090G>A in the genomic sequence, resulting in D364N mutant protein. As the relevant PAM sequence cannot be changed without codon change, a silent mutation in the seed sequence was introduced to prevent Cas9 from cleaving the ssODN itself at position 5 of the seed sequence, since mismatches at position 4-6 are likely to significantly reduce the Cas9 cleavage activity [21]. An additional silent mutation, disrupting an ApaI recognition site, was introduced for easy molecular genotyping using restriction enzymes. Homologous arms of ~100bp were used. For *tin*, the introduced 7bp mutation, AGACACG>CTCATGT, has been demonstrated to reduce *tin* reporter expression in *Drosophila* [16]. Since this change already mutated two PAM nucleotides, no additional change was required. Homology arms of 60bp were used. Homologous arms sequence was based on the reference genome sequence relative to the DSB site, based on the gRNA sequence (3 nucleotides upstream of the PAM sequence).

The designed ssODN is comprised of two homology arms flanking the region to be edited. The orientation of the ssODN strand must be considered, as it must be complementary to the strand that initiates repair [22, 23]. The ssODN should be the reverse complement (anti-sense strand) if the Cas9 cleavage site is upstream of the desired modification site. However, the ssODN should match the sense strand if the Cas9 cleavage site is downstream of the targeted modification site, as is the case for *Nup107* mutation. Full sequences of the designed ssODN are provided in Table 2.

**Table 2:** Sequences for ssODN design. PAM is underlined, gRNA DSB site is indicated by a slash (/), and the introduced mutations locations are indicated in **BOLD**

	***Nup107***	***tin***
Locus	Chr2L (-)	Chr3R (+)
Genomic sequence	GCCGATTCCAAGAACTATGACGAGTACAGCCGCGCGACGGCGGGTGTCTTCTCCGGCCACTTGGGCTCGCTGAAAACCCTTTTGCACAGCAACTGGCAC**G**ATTTGCTCTGGGC**C**CA**T**C/TGAAGGTGCAGATCGACATCCGTGTGGAATCGGAGATACGCGGCTGCTGCCTCAAAAACTACCAACCGATGCCCGATGATT	TGAGGCGGTCTGGGGTGAAAGGAGTACGCGTTCAGTACCAAAATCGAGCTGACAAATTGC**AG/ACACG**GTCGAGTGCGCATCGGAACGGCGCTTTACGGCTTACGGGTTACGGACTACTGATTGCCGATTACGGGC
gRNA target	TTTGCTCTGGGCCCATCTGAAGG	GAGCTGACAAATTGCAGACACGG
Designed ssODN	GCCGATTCCAAGAACTATGACGAGTACAGCCGCGCGACGGCGGGTGTCTTCTCCGGCCACTTGGGCTCGCTGAAAACCCTTTTGCACAGCAACTGGCAC**a**ATTTGCTCTGGGC**t**CA**c**CTGAAGGTGCAGATCGACATCCGTGTGGAATCGGAGATACGCGGCTGCTGCCTCAAAAACTACCAACCGATGCCCGATGATT	TGAGGCGGTCTGGGGTGAAAGGAGTACGCGTTCAGTACCAAAATCGAGCTGACAAATTGC**ctcatgt**GTCGAGTGCGCATCGGAACGGCGCTTTACGGCTTACGGGTTACGGACTACTGATTGCCG

### Crossing Process and Phenotypic Outcome

**Fig. 2:**
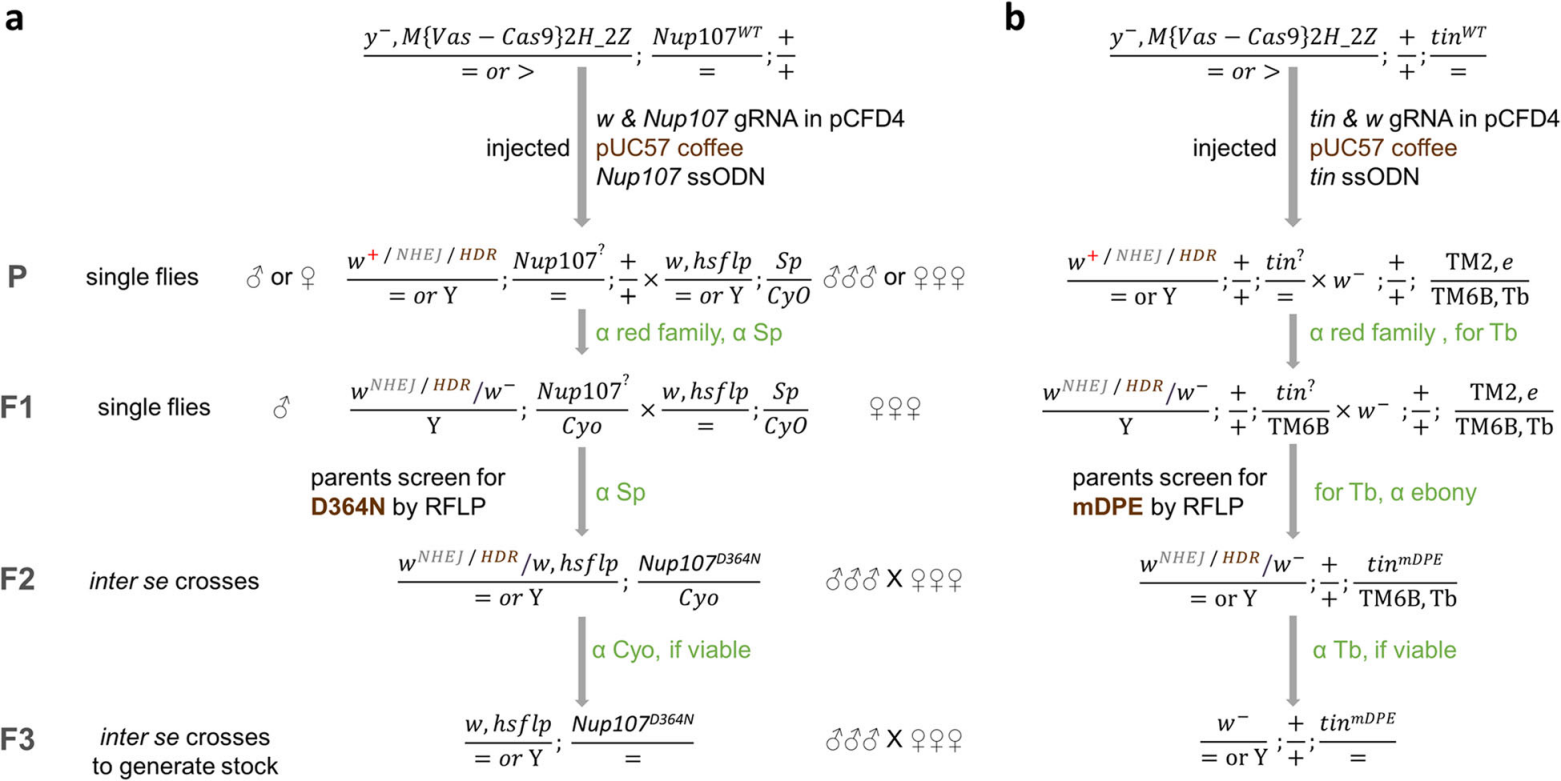
Detailed crossing schemes for chromosome 2 and 3. Detailed crossing schemes followed to generate **a***Nup107*^*D364N*^ and **b***tin*^*mDPE*^ mutant strains. The relevant balancer strains are indicated for each chromosome, as well as the selection criteria in each generation. The injected flies harbor the mutation in the germ cells, and therefore no phenotype is expected in the hatched P flies. Note that the genotype shown for the P flies represents their germ cells and not their soma. Final strains are expected to be either homozygote or heterozygote for the mutation, based on its lethality. In crosses where the P founder fly was a male, the family eye color was determined based only on the females’ eye phenotype, as all F1 progeny males carry the w^−^ allele found on the maternal X chromosome

Parental (P) flies were obtained following injection of the DNA into y^1^, M {vas-Cas9} ZH-2A embryos (Additional file 1: Figure S1) [8]. The crossing scheme that was followed is described in Fig. 2.

Generally, each single parental (P) fly (hatched flies from the injected embryos) was crossed with either virgin females or young males carrying the relevant balancer chromosome. Single F1 male flies from each of the coffee and/or white colored-eyes broods (which carry a mutagenized chromosome in *trans* to the balancer) are then crossed to a balancer stock to generate F2 males and females carrying the same mutagenized chromosome. When F2 progeny flies are crossed to each other (inter se crossing), a third of the F3 progeny are expected be homozygous for the mutagenized chromosome, if viable.

**Fig. 3:**
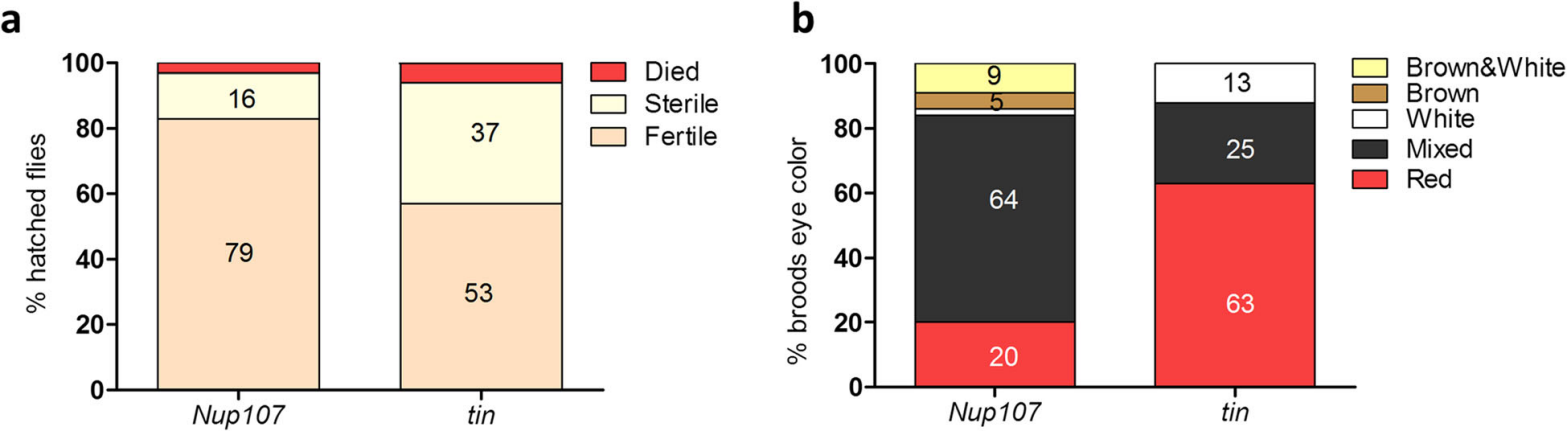
Occurrence for the co-CRISPR strategy generated stocks. **a** The prevalence of fertile, sterile and dead parental (P) flies are presented as percentages of hatched flies. **b** Percentage of the different F1 phenotype eye color group outcome, out of fertile crosses. Numbers for both *Nup107*^*D364N*^ (chr2) and *tin*^*mDPE*^ (chr3) transformants are depicted

It is important to note that P flies have the Cas9-induced modification only in the *germ cells*, so no apparent eye-color phenotype was observed. Most hatched P flies survived and were fertile, as described in Fig. 3a.

Once the F1 flies started to hatch, we have monitored the emerging flies for eye color. Most of the broods presented either red or mixed (red+ white+ coffee) eye color. We selected only white and/or coffee-eyed broods (*i.e.*, non-red family), since they indicate efficient gRNA expression and cleavage by Cas9 at the *white* locus. However, either brown-only (*Nup107*) or white-only (*tin*) broods were identified to be HDR-positive in the relevant locus. The distribution of the detected F1 eye-color outcome is summarized in Fig. 3b.

For each selected F1 brood, single male flies were individually crossed to balancer flies of the opposite sex, thus ensuring the established stocks originate from a single repair event. For all fertile crosses, the founder fly was molecularly tested for the desired recombination event (see relevant methods section). Only HDR-positive flies were kept further and inter se crossed in order to establish a “clean” stock. Since we had no information regarding the lethality of the introduced mutations in *Drosophila*, hatching flies were closely monitored both phenotypically and molecularly. Both *Nup107* and *tin* mutations turned to be viable, and therefore the established final fly stocks are homozygous for the mutations.

Additional notes regarding the crossing process are provided in the methods section.

### Molecular Verification and Phenotypic Examination of the Generated Transgenic Flies

**Fig. 4:**
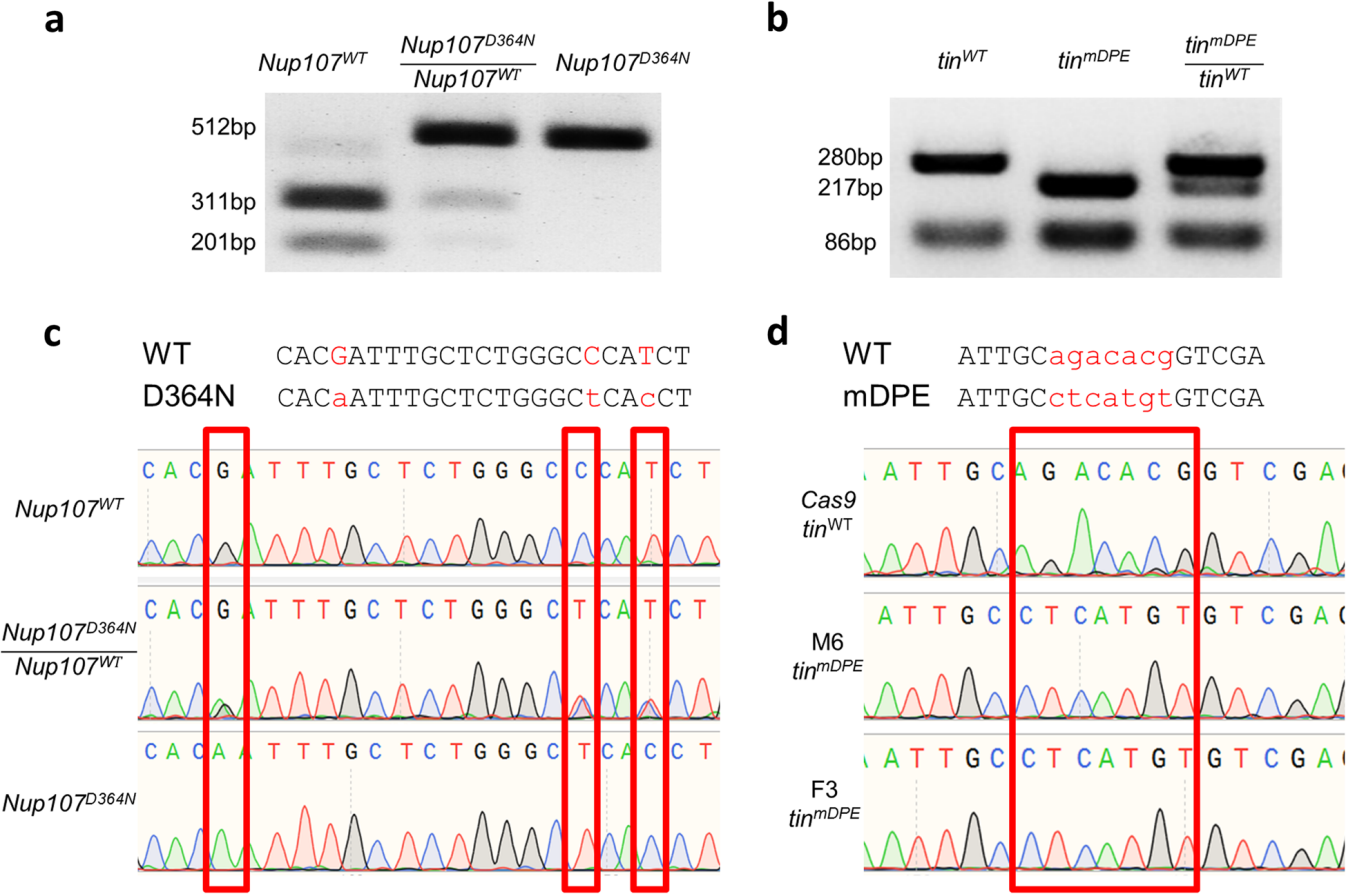
Molecular validation of the generated flies. Molecular confirmation of the genomic mutations used to generate **a***Nup107*^*D364N*^ and **b***tin*^*mDPE*^ transgenic flies. RFLP screening was used to rapidly detect the relevant strains to be further screened. Final strains were validated using Sanger sequencing, as compared to WT flies, for both **c***Nup107*^*D364N*^ and **d***tin*^*mDPE*^

Through the generation of the flies, it is very important to molecularly screen for the desired mutation. Since the designed mutations change restriction enzyme recognition site, restriction fragment length polymorphism (RFLP) was used to efficiently monitor the emerging flies. Using properly designed RFLP, the WT, heterozygotes and homozygote alleles are readily distinguished (Fig. 4a, b), allowing the rapid screening of numerous F1 founder flies. The results of dozens of flies can be detected within the same day, without the need to wait a couple of days for Sanger sequencing results. This alleviates the work load associated with keeping unnecessary flies and thus is strongly recommended. Importantly, in our experience RFLP results always match the Sanger sequencing from the same flies (Fig. 4).

It is of note that for regular screening, involving amplicons of ~500bp, the DNA crude extraction procedure is recommended. However, the resulting genomic DNA preparation still contains high salt concentrations that will interfere with the generation of large PCR fragments. Hence, for amplicons longer than 1kb, we recommend using a specialized genomic DNA extraction kit.

During the generation of the F1 stocks, and more importantly, after establishment of the final stocks, we genotyped the flies using Sanger sequencing. Manual examination of the chromatograms was carried out in order to determine the genotypes of the flies. Heterozygotes were easily detected in the crossing process using this approach. The 80-150bp region surrounding the ssODN ends was also sequenced, and the absence of genomic abnormalities was verified.

**Fig. 5:**
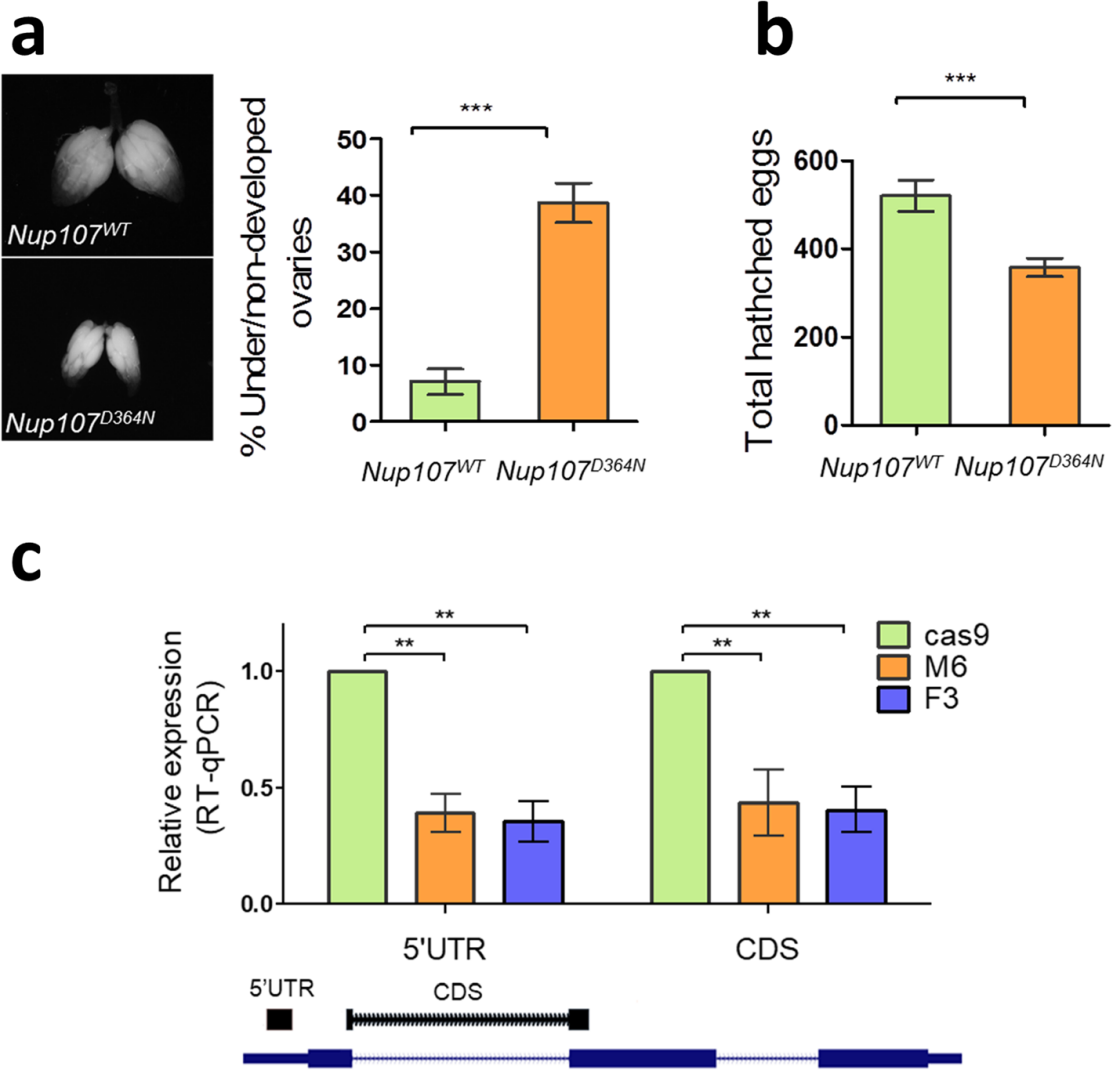
Phenotypic examination of the generated flies. *Nup107*^*D364N*^ mutant flies exhibited **a** ovarian dysgenesis and **b** reduced progeny, as compared to WT control flies. **c***tin*^*mDPE*^ transgenic flies show reduced endogenous *tin* RNA levels at 2-4 h after eggs laying, as measured by RT-PCR. The location of the amplicons is depicted relative to the *tin* gene. Student’s T-test was used to calculate statistical significance of the result, ***p < 0.0002 **p < 0.01

After establishment and verification that the final strains contain the correct genotype, we proceeded to examination of the phenotypic outcome. For *Nup107*, we observed small and under developed ovaries in 38% of *Nup107*^*D364N*^ flies, as compared to 7% in WT control (n=155 and 54 flies, respectively; Fig. 5a). When examining egg production and hatching for *Nup107*^*D364N*^ female flies, we detected lower egg production per day, as compared to WT female controls (36 eggs vs. 52 eggs hatched every day per fly, respectively, Fig. 5b). No other morphological defects were observed (data not shown).

For *tin*, we examined whether endogenous RNA levels in the developing embryo were affected by mutation of the DPE motif, similar to what has been reported in cell culture. Indeed, we were able to detect a reduction in endogenous *tin* RNA levels in the mutant strains, as compared to the WT, at 2-4h of embryonic development (Fig. 5c). This effect was demonstrated using two different primer sets, targeting either the 5’UTR region downstream of the mutation, or the first exon-exon junction.

Taken together, the above results exemplify the importance of introducing point mutations in the endogenous genomic context, allowing the analysis of the in vivo contribution of specific nucleotides to distinct functional regions.

## Discussion

A ssODN template is the ultimate choice for introducing point mutations, as well as small sequences such as protein tags. However, screening for a single nucleotide change is technically challenging and labor-intensive, requiring multiple molecular screens of single flies. To generate a stock, each recombination event must be handled separately, down to the level of each gamete. This usually involves numerous single crosses, greatly increasing the number of flies to manage. Utilizing the strength of *Drosophila melanogaster* genetics and coupling the desired HDR-introduced change with a visible marker alleviates much of the burden associated with molecular screening. Essentially, the fly screening is performed by visual examination, and therefore only relevant flies containing the desired mutation are further crossed.

However, direct addition of the visible marker to the examined locus will “contaminate” the genomic context and is therefore counterproductive, especially when considering point mutations. Currently, two main approaches exist: scarless genome editing ([9], https://flycrispr.org/scarless-gene-editing/) and the co-selection strategy, using either eye or body color [8, 24]. While the scarless strategy enables the visualization of the HDR event by first integration followed by the removal of a visible marker, the co-conversion strategy relies on a repair event at another, unrelated, locus occurring simultaneously to the desired change. The scarless genome editing approach depends on a plasmid DNA donor, thus the chances of donor integration into the examined locus are increased [9]. Indeed, we (Juven-Gershon lab) observed a 100% rate of donor integration when using this approach, across 3-4 independent lines in two distinct loci. Therefore, the ability to introduce point mutations using ssODN and easily screen for them as proposed by the co-conversion strategy is very important. Notably, a negative co-selection strategy has been reported, where a dominant female sterile allele is successfully edited in order to produce offspring, thus enriching the positive transformants ratio among the screened population [25].

We described the successful application of *white* co-conversion using a ssODN template, instead of the originally described dsDNA plasmid. We used the technique to successfully introduce point mutations in two distinct chromosomes.

Although the successful application of *white* co-conversion has recently been reported [26], to our knowledge, this is the first report of using the approach in combination with a ssODN template. In addition, we provide detailed comprehensive guidelines for designing, performing and screening processes. These guidelines are based on the experience obtained in two distinct labs, and should thus be easily reproducible in other labs. We also provided detailed crossing schemes that can be adapted to other genes of interest. The main disadvantage of using ssODNs is its size constraint, usually restricted to 200 nucleotides, which limits its use for several-bp long mutations. Recently, however, the use of longer ssDNAs (~1000 nucleotides) as homology donors was demonstrated in vivo in both mice [27] and flies [28], and may become common practice over time.

Using the flies generated by these protocols, we were able to demonstrate the in vivo importance of the respective mutations. For the *Nup107* gene, c.1090G>A (p.D364N) mutation was confirmed to affect ovarian development, observed as underdeveloped ovaries and reduced numbers of hatched eggs in the homozygote flies compared to WT flies (Fig. 5a, b). For the *tin* gene, the regulatory role of the DPE core promoter motif was demonstrated in the developing *Drosophila melanogaster* embryo (Fig. 5c), thus providing major evidence for core-promoter-based transcriptional regulation during embryonic development.

## Conclusions

In conclusion, the CRISPR technology using ssODN donors is a highly efficient technique as compared to CRISPR using dsDNA donors or other genetic manipulations for small insertions or deletions. In this paper we combined the *white* co-conversion screening approach with a ssODN template to successfully generate transgenic fly strains using CRISPR. Using this approach, we were able to introduce very specific point mutations into the endogenous genomic loci without apparent genomic scars, in a relatively simple, fast, easy and efficient method. We provide detailed guidelines, recommendations and crossing schemes that can be used towards the generation of other CRISPR transgenic flies. Lastly, using this strategy we identified distinct phenotypes associated with the newly-generated genotype as compared to the WT strain, thus exemplifying the importance of investigating the in vivo function of point mutations.

## Methods

The described methods include the description of the work, along with general guidelines that can be used for implementing the strategy for other genes of interest.

### Fly Maintenance and Stocks Generation

For all described procedures, flies were raised, maintained and crossed on standard cornmeal yeast extract media (cornmeal, yeast, molasses, and agar) at 25°C.

Target loci of vas-Cas9 (y^1^, M {vas-Cas9} ZH-2A)) from Rainbow Transgenic Flies, Inc. (Camarillo, CA) were sequenced prior to sgRNAs design to avoid SNPs and injected. *y,w,hsflp; Sp/CyO;* or w^-^*;;TM2/TM6B* balancer lines, depending on the relevant chromosome, were used for crossing with the injected flies.

Detailed crossing schemes are shown in Fig. 2; the sequences used to construct the flies are listed in Tables 1 and 2.

### gRNA Design and Cloning

gRNA for both *Nup107* and *tin* were designed using the FlyCRISPR algorithm (http://targetfinder.flycrispr.neuro.brown.edu/ [3];) with default parameters. Both gRNAs (per gene) were cloned into pCFD4d ([7], Addgene #83954) using the following methods:

1. Gibson assembly reaction [18] was used to clone *Nup107* gRNA construct.

- Primers were designed to span 30 bp homology to pCFD4d and ordered as desalted, non-HPLC purified oligos (IDT or HyLabs).
- PCRBIO HiFi Polymerase (PCRBiosystems, PB10.41) was used to amplify the *white* (w) and *Nup107*^*D364N*^ (*Nup*) gRNAs fragments using undigested pCFD4d plasmid as a template.
- 2 μg of pCFD4d plasmid were linearized using BbsI restriction enzyme (NEB R3539) for 2 h at 37 °C.
- The generated DNA was analyzed by running 5 μL of the reaction on an agarose gel. The PCR fragment and the digested plasmid were gel-purified using ZYMO’s gel purification kit (ZYMO D3100).
- 10 μL of total DNA (0.02–0.5 pmol containing all fragments) was added to 10 μL of Gibson mix (NEB E5510S) and incubated at 50 °C for 1 h.

2. Transfer PCR (TPCR) method [19, 20] was used to clone the *tin* targeting construct.

- A PCR reaction was setup using Q5 Polymerase (NEB M0491), following the manufacturer’s protocol with the following changes:
  The concentration of each primer was 1μM (diluted 1:10 from a 10μM stock). The pCFD4d plasmid template was diluted to a final concentration of 10ng/μl, and 2μl (20ng) were used for the reaction. Cycling conditions are listed in Table 3.
- Following the completion of the PCR reaction, 1 μl of DpnI (NEB R0176) were added to the PCR reaction, and the reaction was incubated at 37 °C for 1 h to eliminate the original (methylated) template.

**Table 3:** TPCR conditions for cloning both gRNA into pCFD4d plasmid. The denaturation-annealing-elongation cycle was repeated 30 times

Temperature (°C)	duration	
98	30 s	Initial denaturation
98	10 s	Denaturation
61	1 min	Annealing
72	7 min	Elongation
72	7 min	Final extension
10	–	Storage

5μl of the *Nup107* or *tin* constructs were transformed into competent *E. coli* bacteria and plated on Ampicillin-containing plates using standard heat-shock procedure (42°C for 45-90 sec).

The sequences of the generated plasmids were verified using a pCFD4d sequencing primer (Table 1), and midiprep-grade DNA was prepared using QIAGEN Plasmid Midi Kit (QIAGEN 12143).

### ssODN Resuspension

The designed ssODN sequences (Table 2) were ordered as a 4 nmole Ultramer® DNA Oligo (IDT), and resuspended in ultra-pure water (UPW) to a final concentration of 1μg/μl upon arrival.

### Injection Conditions

We have used the injections services of Rainbow Transgenic Flies (https://www.rainbowgene.com/).

Injected flies are Vas-cas9 (305). Full genotype is y^1^, M {vas-Cas9} ZH-2A generated by recombining y^1^, M {vas-Cas9} ZH-2A, w^1118^ (Bloomington 51323) with Oregon-R (described in [8]).

The injection mix containing 250ng of each pCFD4d and pUC57-white [coffee] plasmids (in TE), together with 500ng of the ssODN (in UPW), was injected into *Drosophila* embryos that express transgenic Cas9 under a germline specific promoter (vas-Cas9, Additional file 1: Figure S1).

pUC57-white [coffee] plasmid (Addgene plasmid 84006) was prepared as a high-quality midiprep-grade DNA.

Transformants were shipped as injected larvae and have reached the lab before hatching. Larvae were treated well to ensure large numbers of hatched flies.

### Crossing Schemes

Crosses were set according to the excellent guidelines described in [17].

The following notes are arranged in order of the crossing scheme stages, as provided in Fig. 2. During all stages, virgins were immediately collected, as soon as the flies started to hatch, otherwise no crosses could be set.

#### Preparing for the Injected Flies/ P Flies

- When the DNA was sent for injection, we started expanding the relevant balancer populations, in order to have enough virgins once the injected flies will be ready.
- Once the injected larvae start to hatch, we began collecting virgins and males separately.
- As the parental (P) flies should have the Cas9-induced modification in the *germ cells*, no apparent eye-color phenotype was expected.
- Each hatched fly was individually crossed to 3 balancer flies of the opposite sex. Crosses were set according to the total amount of hatched flies.
- The crosses were labeled as M1, M2,../ F1, F2, … to represent the original P founder.
- When setting the crosses, we always used fresh food sprinkled with yeast.

#### F1 Flies

- Once the F1 flies started hatching, the eye color of the emerging female flies was carefully recorded, looking for either white and/or coffee broods (non-red group). Individual flies from mixed broods could theoretically be crossed as well. However, in our experience, molecular screening revealed these flies do not harbor the desired genomic alteration.
- Since each fly represents a different gamete of the P fly (each F1 is an independent event), each F1 fly was crossed to a balancer fly of the opposite sex. This ensured that the established stock originated from a single repair event. For each selected brood, we crossed at least 10 F1 individuals.
- Only after enough progeny were detected (based on larvae), the founder F1 fly was tested for the desired recombination event using molecular screening (see the relevant section).
- Only HDR-positive flies (based on the RFLP analysis) were subjected to subsequent analysis.

#### F2-F3 Flies

- In order to establish a “clean” stable stock, several more crosses were required. Since at this point all HDR-positive flies originated from a single event, it was acceptable to cross siblings (inter se crosses) (Fig. 2).
- Depending on the lethality of the specific mutation, homozygote flies may or may not be detected. Thus, hatching flies were carefully monitored, both phenotypically and molecularly.
- Note that the emerging flies can be crossed to either *lacZ* or fluorescent balancers if necessary.
  Possible stocks are listed here
  https://bdsc.indiana.edu/stocks/balancers/balancer_lacZ.html
  https://bdsc.indiana.edu/stocks/gfp/fluor_balancers.html

### Molecular Genotyping

NucleoSpin® DNA RapidLyse kit (MACHEREY-NAGEL, 740100) was used for extraction of genomic DNA for amplicons larger than 1kb. For sequencing, the resulting DNA fragment was gel-purified (using commercially-available kits) and sent to Sanger sequencing.

#### Crude Genomic DNA Extraction

1. The number of flies to be screened was determined.
  Homogenization buffer (10 mM Tris-HCl pH 8.2 1 mM EDTA, 25 mM NaCl in DDW) was prepared, and 200 μg/ml Proteinase K was added just before use.
2. 50 μl of Proteinase K-containing homogenization buffer was aliquoted to numbered 1.7 ml tubes.
3. A single anesthetized fly was added to each tube. Note that when the final stock has been generated, it is possible to pool 5 flies per vial. In this case, the homogenization buffer needs to be scaled up accordingly.
  Any relevant information about the fly in the tube was carefully recorded, as this fly will no longer be available.
4. Each fly was squished using a plastic pestle or an autoclaved 20 μl tips.
  It is of note that pestles can be reused if stored in ~10% bleach. Make sure to carefully wash the bleach before each use. In case of reusing pestles, it is recommended to perform a PCR control on a solution with pestle only, in order to monitor for DNA contamination.
5. The squished flies were incubated at 37 °C for 20–30 min.
6. Proteinase K was inactivated by incubation at 95 °C for 2 mins.
7. The tubes were centrifuged for 7 min at 18,000 g.
8. ~1 μl of the supernatant is used per 10 μl PCR reaction.

Note that the resulting genomic DNA can be stored at 4°C for months.

### RFLP

It is important to perform both negative and positive controls, in order to rule out reagents contamination and ensure proper interpretation of the results, respectively.

Crude genomic DNA was amplified using the relevant primers (Table 4) and PCRBIO Taq DNA Polymerase (PCRBiosystems, PB10.13). Reactions of 10μl were routinely used, assembled and ran according to the manufacturer’s protocol. The restriction enzyme (ApaI or NlaIII) was added to the PCR products. Digestion was performed at 37°C for at least 1hr. Products were resolved on a 1.5% agarose gel (an example is shown in Fig. 4a, b).

**Table 4:** Primer sequences used and amplicon sizes of *Nup107* and *tin* for RFLP

	***Nup107***	***tin***
Forward primer	GAGCAGAATGTCTCGGTGCT	ATTCCGATGCTGTGCTGTGATTG
Reverse primer	AGGAAGAGCACTATGTGGGC	TTAAATAAGTCCAACAATTTGCC
PCR product length (bp)	512	498
Digestion enzyme (recognition site)	ApaI (GGGCC^C)	NlaIII (CATG^)
Digested product size (bp, WT/mutant)	311 + 201/512	412 + 86 / 348 + 86 + 64

### RNA Extraction and Real Time PCR Analysis

2-4h embryos were collected and aged at 25°C. Two independent mutant strains (F3, M6) and the WT strain (Cas9) were collected and processed in parallel. Collected embryos were dechorionated in bleach for 2 minutes, and then transferred to an 1.7 ml tube. Embryos were overlaid with TRI Reagent (Sigma-Merck T9424), squished using a pestle and stored at -80°C until RNA extraction. Total RNA was extracted according to the manufacturer’s protocol. 1 μg RNA was further used for cDNA synthesis (qScript cDNA Synthesis Kit, Quantabio 95047). Quantitative PCR using SYBR green (qPCRBIO SyGreen Blue Mix, PCRBiosystems, PB20.12) was performed using a StepOnePlus Real-Time PCR machine. Control reactions lacking reverse transcriptase were also performed to ensure that the levels of contaminating genomic DNA were negligible. Transcript levels were analyzed by ΔΔCT method using RpII18 as an internal control (FW- AGTGATGGATGATGCGGACT, RV- ATGATCTCGATGTTGTCCGC). Each sample was run in triplicates. The results represent the average of 4 biological replicates.

### Ovarian Development Assay

Ovaries were extracted in ice-cold PBS from 1–5 days old yeast-fed adult females, which had been kept in the company of males. Flies were kept under standard conditions with yeast pellet for at least 24 hrs prior to dissections using fine biological tweezers. The overall size and developmental level of the ovaries were tested under binocular.

### Fertility Assay

Ten virgin female and 10 young male flies from the respective maintenance bottles of the Nup107 groups (WT or mutant flies) were recombined into progeny breeding cages. The flies in these cages were maintained on agar plates with yeast pellet and were given fresh food every day. The number of hatched and non-hatched eggs from each bottle was recorded. Data are represented as the mean of six independent experiments compared to the WT control (Cas9 flies without the mutation).

## Supplementary information

**Additional file 1: Figure S1.** Schematic representation of the injection mix used to introduce the genomic modifications. A plasmid containing the *white [coffee]* donor, ssODN targeting the *Nup107*^*D364N*^ or *tin*^*mDPE*^ area of interest (199 bp or 126 bp, respectively), and pCFD4 plasmid containing gRNA for *w* gene and *Nup107*^*D364N*^ or *tin*^*mDPE*^ were co-injected into *Drosophila* syncytial blastoderm of embryos that express transgenic Cas9 (vas-Cas9).

## Data Availability

All data generated or analysed during this study are included in this published article and its supplementary information files.

## Abbreviations

CRISPR: clustered regularly interspaced short palindromic repeat
DPE: downstream core promoter element
DSBs: double strand breaks
dsDNA: double-strand DNA
gRNA: guide RNA
HDR: homology-directed repair
NHEJ: non-homologous end-joining
*Nup107*: *Nucleoporin107*
P: parental
PAM: protospacer adjacent motif
RFLP: restriction fragment length polymorphism
ssODN: single-strand oligodeoxynucleotide
*tin*: *tinman*
*w*: *white*
XX-OD: XX ovarian dysgenesis
