# Supplementary figures and images for: Efficient In Vivo Introduction of Point Mutations Using ssODN and a Co-CRISPR Approach
Source: Biol Proced Online. 2020 Jul 14;22:14. doi: 10.1186/s12575-020-00123-7 (PMC7362497; doi:10.1186/s12575-020-00123-7)

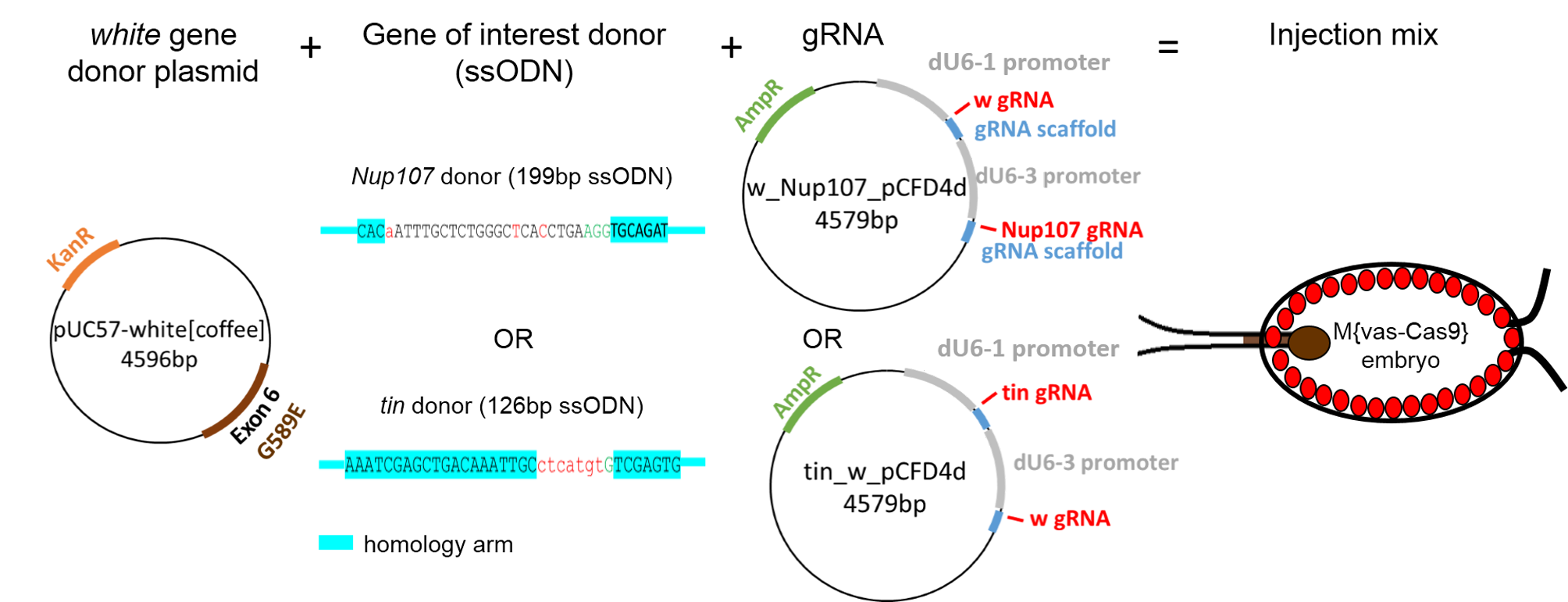

Supplement: Supplementary file 1 — Additional file 1: Figure S1. Schematic representation of the injection mix used to introduce the genomic modifications. A plasmid containing the white [coffee] donor, ssODN targeting the Nup107D364N or tinmDPE area of interest (199 bp or 126 bp, respectively), and pCFD4 plasmid containing gRNA for w gene and Nup107D364N or tinmDPE were co-injected into Drosophila syncytial blastoderm of embryos that express transgenic Cas9 (vas-Cas9). [file 12575_2020_123_MOESM1_ESM.tif]
